# Lattice Model for Influenza Spreading with Spontaneous Behavioral Changes

**DOI:** 10.1371/journal.pone.0083641

**Published:** 2013-12-23

**Authors:** Annalisa Fierro, Antonella Liccardo

**Affiliations:** 1 Consiglio Nazionale delle Ricerche (CNR) - Institute Superconductors, oxides and other innovative materials and devices (SPIN), Napoli, Italy; 2 Physics Department, Università degli Studi di Napoli “Federico II”, Napoli, Italy; 3 Istituto Nazionale Fisica Nucleare (INFN) - Sezione di Napoli, Napoli, Italy; Fondazione Bruno Kessler, Italy

## Abstract

Individual behavioral response to the spreading of an epidemic plays a crucial role in the progression of the epidemic itself. The risk perception induces individuals to adopt a protective behavior, as for instance reducing their social contacts, adopting more restrictive hygienic measures or undergoing prophylaxis procedures. In this paper, starting with a previously developed lattice-gas SIR model, we construct a coupled behavior-disease model for influenza spreading with spontaneous behavioral changes. The focus is on self-initiated behavioral changes that alter the susceptibility to the disease, without altering the contact patterns among individuals. Three different mechanisms of awareness spreading are analyzed: the local spreading due to the presence in the neighborhood of infective individuals; the global spreading due to the news published by the mass media and to educational campaigns implemented at institutional level; the local spreading occurring through the “thought contagion” among aware and unaware individuals. The peculiarity of the present approach is that the awareness spreading model is calibrated on available data on awareness and concern of the population about the risk of contagion. In particular, the model is validated against the A(H1N1) epidemic outbreak in Italy during the 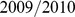 season, by making use of the awareness data gathered by the behavioral risk factor surveillance system (PASSI). We find that, increasing the accordance between the simulated awareness spreading and the PASSI data on risk perception, the agreement between simulated and experimental epidemiological data improves as well. Furthermore, we show that, within our model, the primary mechanism to reproduce a realistic evolution of the awareness during an epidemic, is the one due to globally available information. This result highlights how crucial is the role of mass media and educational campaigns in influencing the epidemic spreading of infectious diseases.

## Introduction

Understanding the way people interact with each other is one of the key ingredients for the comprehension of the epidemic spreading process. Most epidemiological models are indeed obtained by combining a transmission model with a socio-demographic model, based either on a microscopic approach, as in the IBM models [1]–[4], or on data informed approaches, as those that make use of large scale surveys on social contacts [5], [6]. Typically, the socio-demographic structure and the kind of interactions among individuals are assumed to remain unchanged during the epidemic. This assumption, although useful to outline the problem, is clearly unrealistic. During an epidemic outbreak, behavioral changes occur, due to both institutional measures (vaccination campaign, school closure, travel restrictions, etc.) and self-initiated measures directed to reduce the risk of contagion. The literature that treats institutionally induced behavioral changes, is extensive (e.g. [7]–[13]). The self-initiated behavioral changes are instead more difficult to be identified and modeled. On the other hand, it is a matter of fact that, as soon as individuals perceive the risk of contagion, they tend to be more cautious in their social contacts, assuming an adaptive behavior. In so doing, they can alter the socio-demographic structure, increasing the social distancing, and/or avoiding those kind of contacts that may enhance their exposition to the virus. These mechanisms significantly interfere with the progression of the epidemic. As a consequence, any realistic epidemic model cannot disregard the interplay between human behavioral changes and the spreading of the epidemic itself.

In recent literature, there are some interesting works, which concentrate on the effect of risk perception [14]–[17]. Evolutionary game theory is often invoked to model the effect of the human response to the risk of contagion. In this approach, the behavior is understood as a strategy with a specific pay-off and the individual behavioral choice, in front of the risk of contagion, is dictated by a comparison among the pay-offs of different strategies. This approach has been adopted to investigate both the response to voluntary vaccination programs [18]–[21] and the effect of self-prophylaxis measures or reduction of physical contacts, which alter the susceptibility or the exposition to the infection [22], [23]. In Ref. [24], the self-initiated behavioral changes are classified with respect to (i) the source of the information that induces the adaptive behavior, which can be either local or global; (ii) the type of information, which can be based on the effective number of infected individuals (prevalence based) or independent of the disease prevalence (belief based); (iii) the effects of the behavioral changes, which may include modifications of the epidemiological state of the individual (e.g. vaccination campaigns), modifications in the transmission parameters (e.g. prophylaxis procedures), modifications of the contact network structure (e.g. increased social distancing).

In Ref.s [25], [26], we have recently developed a stochastic SIR model for influenza spreading on a lattice, which represents the dynamic contact network of individuals. An age distributed population is placed on the lattice and moves on it. The displacement from a site to a nearest neighbor empty site, according to certain mobility rules, allows individuals to change the number and identities of their contacts. The model is based on the social contact hypothesis, i.e. it assumes that the spreading of an infectious disease is mainly regulated by the average age-dependent contact patterns of individuals, thus the dynamics model is designed with the aim to reproduce those patterns [6]. A simple SIR transmission model, with a nearest neighbor interaction and some very basic adaptive mobility restrictions, complements the model. The model is validated against the age-distributed Italian epidemiological data for the influenza A(H1N1) during the 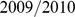 season, with sensible predictions for the epidemiological parameters. In particular, the model reproduces the epidemiological data during the epidemic peak for all the age-classes. Deviations are found in the descendant phase, mainly for young people. In Ref. [26], it was suggested that such deviations could be partly due to self-initiated behavioral changes, which might have had a non-negligible impact on the progression of the A(H1N1) pandemic in Italy. During the peak, indeed, the spread of awareness and fear, due in particular to a strong media campaign and to the information published by public health authorities, resulted in individual behavioral changes, able to reduce the spreading of the disease. In Ref.s [25], [26], some disease adaptive restrictions, that alter the contact network of individuals during the spreading of the epidemic, were already included. In particular, we imposed that an infected individual stops moving as soon as symptoms appear, and that nobody can have contact with her/him during the symptomatic phase, except those that were already her/his nearest neighbors at the stop time, representing the family members in our simplified model. In the framework of the above lattice model for influenza spreading, here we focus on the impact of self-initiated behavioral changes that reduce the susceptibility to the disease, altering the transmission parameters, as for instance increased hygienic measures and use of face masks.

Awareness and fear of the risk of contagion may spread among the population in many different ways. Here, we concentrate on three different mechanisms: 1) the local spread of awareness due to the presence in the neighborhood of infective individuals, that renders neighbors aware about the risk of contagion; 2) the global spread of awareness due, for instance, to the news published on the mass media and to institutional educational campaigns; 3) the local spread of awareness occurring through a mechanism of “thought contagion” [27] similar to that of infectious diseases. Different scenarios that correspond to different combinations of these awareness mechanisms are analyzed. Let us emphasize that the first mechanism for awareness spreading is governed by the epidemic spreading itself, while the second and third mechanisms require the introduction of further parameters to establish how the number of infected individuals influences the global spreading of concern about the disease, and to fix the probability of thought contagion. In order to fix these parameters, we adopt a data-informed strategy. In particular, we use data on risk perception gathered in Italy by the behavioral risk factor surveillance system [28]. These data allow to construct a cross-check strategy that put together epidemiological data and awareness data.

Summarizing, the main motivation of the present paper is to analyze the interplay between the epidemic spreading of an infectious disease and the spontaneous behavioral changes of the population in response to the epidemic itself. In so doing, we adopt a phenomenological approach, by testing our model both on awareness and epidemiological data. We show that a better agreement between simulated and gathered data on awareness leads to a better agreement also for the epidemiological data. Furthermore, we show that the crucial mechanism for reproducing a realistic trend for the awareness spreading among the population, is the one triggered by the mass media.

## Materials

### Epidemiological Data

Influnet is the influenza surveillance system in Italy, coordinated by the Ministry of Health in collaboration with the Istituto Superiore di Sanità (ISS), the Interuniversity Centre for Research on Influenza (CIRI), the Regional Health Departments, the general practitioners and pediatricians and some university laboratories. This system collects and publishes data on the influenza spreading on the entire national territory, since the season 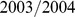, and it is based on nationally organized sentinel networks of physicians with a coverage of at least 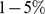 of the population. Because every sentinel physician reports the aggregated weekly number of patients seen with ILI, the reporting rate results to be one per week.

Age-specific influenza-like illness (ILI) incidence data of H1N1 pandemic during the season 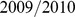 are available in the report [29] with a partition in four age classes (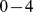, 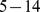, 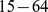, over 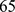 years old). Apart fluctuations, the number of physicians participating to the surveillance during the H1N1 epidemic, and correspondingly the number of patients, is stable during the period between 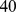-th and 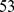-th week, considered for our simulations.

### Data on Risk Perception

The existence of a behavioral risk factor surveillance system in Italy (known as PASSI, Progressi delle Aziende Sanitarie per la Salute in Italia) [30], allowed to analyze knowledge, attitudes and behaviors of the adult population (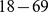 years) regarding the flu pandemic A(H1N1), starting from the peak of the outbreak to the end of the epidemic. In Ref. [28], authors show the trend over time of different indicators, which describe awareness and concern of the population about the risk of contagion. The most relevant indicators, as the perception of high risk of being infected, and the worry about the pandemic, as expected, are decreasing functions of time (dropping roughly from 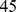 % of the interviewed persons at the peak, to respectively 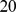
% and 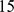
%, three months later). Interestingly, a high percentage of individuals were aware of the main hygienic measures to control the virus spread (the aware people, averaged over the considered period, was roughly 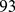
% of the interviewed persons). Thus, this study proves the effectiveness of the Government informative campaign, centered on the adoption of effective hygienic measures for preventing the influenza spreading, and confirms the idea that behavioral changes, directed to the reduction of the susceptibility to the disease, might have had a role in the spreading of A(H1N1) influenza in Italy. According to Ref. [28], it seems instead that social distancing had a definitely minor impact on the epidemic spreading, since the percentage of individuals that in the same period declared to have restricted their activity out of home was on average roughly 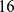
% of the interviewed persons. This justifies our focusing on behavioral changes that induce the reduction of susceptibility of aware people, rather than structural changes of the contact network among individuals.

### Mass Media Coverage

**Figure 1:**
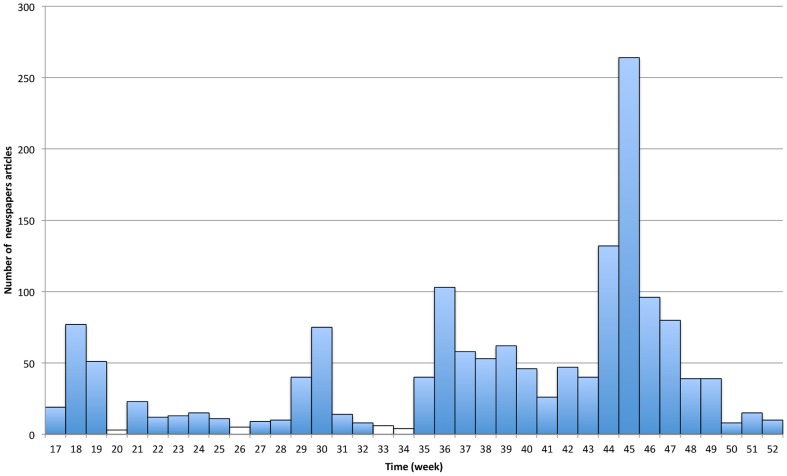
Number of articles, which mentioned the term ‘H1N1’, appearing from week 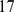 (from April, 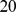
th to April, 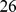
th) to week 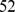 (from December, 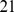
th to December, 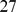
th) on the top four Italian newspapers: *Corriere della Sera, La Repubblica, La Stampa* and *Il Sole 24 ore*.

Italian media devoted intense attention to the epidemic spreading, especially during the peak. In order to have a quantitative understanding of this phenomenon, we performed an analysis of the number of articles, mentioning the term ‘H1N1’, which appeared from week 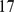 (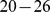 April) to week 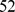 (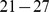 December) on the top four national newspapers: *Corriere della Sera, La Repubblica, La Stampa* and *Il Sole 24 ore*, which cover the 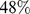 of the overall newspaper circulation in Italy. Data from TV and Radio are not included in the present analysis. The results are plotted in Fig. 1. From the data collected, it emerges that the 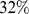 of the overall news, published in the observed time window of 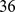 weeks, was concentrated only in 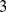 weeks (from week 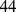 to week 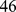), which indeed correspond to the epidemic peak. Moreover, the distribution of the news in Fig. 1 follows a discontinuous path, with the alternation of periods of alarmism and quiet. During the spring (weeks 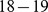), the first laboratory confirmed cases were reported in Europe (first in Spain and UK, and then in many other European countries). The first ascertained case in Italy was reported on the 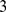-rd of May. At the same time, the OMS upgraded the classification of the H1N1 from Phase 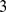 to Phase 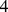 (from small clusters to community level outbreak) on April, 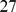
th 2009, and up to Phase 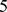 (human-to-human spread of the virus into at least two countries in one WHO region) on April, 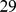
th 2009. These events called the attention of the media on the H1N1, generating a first peak in the number of news during the spring. Other moderate peaks of information were observed during the summer, in correspondence of very specific events (the first death of an Italian individual living abroad, adolescents and young people contracting the infection while they were attending English holidays courses abroad, the announcement of a possible delay in the opening of schools, etc.). However, a systematic presence of a high number of daily news on the H1N1 is observed only in fall, during the outbreak of the epidemic. Furthermore, the analysis of the news published before the school opening (mid of September), shows that they mainly focused on international aspects, rather than local ones, while those published during the peak clearly focused on the local outbreak, and in particular, on the massive spread of the disease among children in schools, and on death cases. In particular, analyzing the content of the news reported by the newspapers, we explicitly checked that the number of news that mentioned all three terms *‘H1N1’, ‘children’* and *‘school’* in the time window from October, 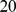
th to November, 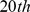 (roughly from week 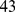 and half to week 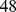 and half) was triple with respect to the one of the previous 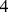 weeks, when schools were already open but the number of cases was still small. A careful analysis of the correlation between media coverage and risk perception, with the inclusion of data on other media (e.g. TV and Radio) will be discussed in a forthcoming paper.

## The Model

In this section, following Ref.s [25], [26], we briefly illustrate the baseline model for influenza spreading. Then, we introduce the self-initiated behavioral changes and discuss the impact on the epidemic spreading.

### Contact Network Dynamics

The model is an attractive lattice-gas on a 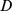 dimensional lattice [25], [26]. The population is distributed on the lattice according to the age group densities of a specific country. In particular, the lattice is occupied by 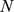 individuals of 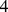 different types, labeled by the index 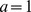, 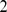, 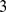, 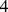, corresponding respectively to the age groups, 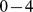, 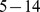, 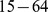 and over 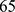 years old. Contacts and transmission of the infection occur only between nearest neighbors on the lattice. Notice that no notion of distance is defined on the lattice, which instead represents the dynamic contact network of individuals. The existence of empty sites allows individuals to change the identities (and eventually the number) of their simultaneous contacts by moving from a site to a nearest neighbor empty site, which corresponds to move from a certain environment/social group to another (e.g. from work to home). The dynamics is governed by an attractive interaction among individuals of the same age class, and the parameters of the model are chosen so that the overall daily number of contacts reproduces the corresponding Polymod data in each age class [6].

In details, defining the nearest neighbor effective number, 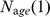, of the individual located at the site 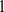, and belonging to a certain age class, as the total number of nearest neighbors belonging to the same age class, one can define the following algorithm. At each step of the dynamics:

1. choose at random an individual (located at the site 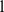), and a nearest neighbor destination site (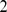), on the lattice. If the site 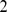 is occupied, another individual is randomly chosen. The probability that the randomly chosen site 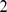 is empty depends on the local occupation density, increasing as the crowding decreases;
2. if the site 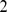 is empty, try to move the individual from the site 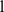 to the site 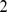 with the probability: 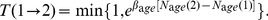
(1)

The movement from the site 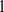 to the site 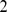 occurs with probability 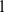, if the number of nearest neighbors in the same age class increases or remains constant, otherwise it occurs with probability 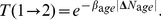
(2)with 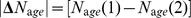. Thus, the probability for an individual to move from the site 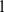 to a randomly chosen nearest neighbor site 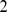 is given by the probability that the site 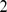 is empty, times 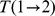, where the first term favors spacing and the second one instead crowding.

The parameters, 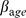, are the age-dependent inverse mobilities, and play the role of assortativity regulator. In our simulations, they are fixed so as to reproduce the data on the number of total (both physical and non-physical) age-dependent daily contacts, furnished by the Polymod large scale survey [6], in which the contact patterns, relevant for infections transmitted by the respiratory or close-contact route, were acquired in 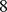 EU countries. For further details on the role of 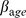 in the dynamics of individuals we refer to Ref. [26].

The model, which we use to simulate the contact network dynamics, was borrowed from the Statistical Mechanics. In that context, lattice models are usually studied using numerical simulations, employing Metropolis algorithm. A lattice-gas is a system of 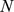 particles distributed on a lattice. Usually, each site of the lattice can be occupied only by 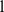 particle and nearest neighbor particles interact via an attractive potential. Here, we generalize this model considering particles belonging to 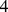 different classes: particles of the same type attract each others with a strength proportional to 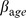, while particles of different types interact only by means of the excluded volume (i.e., a lattice site cannot be occupied by two or more particles simultaneously). Using a Hamiltonian formalism, this model is described in terms of a specific mathematical operator (the Hamiltonian), which gives the energy of the system: 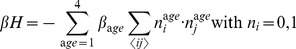
(3)where the sum 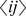 runs over the nearest neighbor site 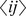, the index 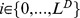 and 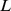 is the linear size of the lattice, 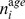 is the age class occupation number of the site 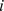, which is 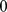, if the site is empty, and 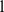, if the site is occupied by one individual of the age class “age”. More details on the Hamiltonian description can be found in Ref[26].

In such a context, the algorithm here adopted, Eq.(1), is obtained as a standard Metropolis algorithm for a Hamiltonian system in the canonical ensemble (i.e., at constant 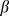).

### Baseline Transmission Model

The previous model for the population dynamic is coupled with a SIR stochastic model, in which each individual can be healthy without immunity (i.e. susceptible, S), infective (I) or healthy with immunity (i.e. recovered, R). An internal degree of freedom for the healthy/infective status (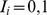) is associated to each individual, and a further degree of freedom for susceptible/immune status (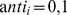) is attributed to healthy individuals. After a potentially contagious contact with an infected nearest neighbor, a susceptible (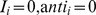) becomes infected (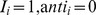) according to her/his specific age class susceptibility, 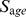.

The infective individual goes through an asymptomatic phase, followed by a symptomatic one. During the epidemic, some disease adaptive rules are over-imposed:

- Infected individuals typically stay at home during the manifestation of symptoms, reducing their contact network essentially to the family. This tendency is implemented in the model by imposing that, at time 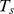 (where 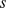 stays for stop or symptoms) after the contraction of the infection, the infected individual stops and does not move until she/he recovers.
- Susceptible individuals tend to avoid contacts with the infected ones during their symptomatic phase. This tendency is implemented by imposing that the empty sites, which are nearest neighbor to symptomatic infected individuals, are interdict. In other words, symptomatic infected individuals can only infect their susceptible nearest neighbors at the stop time, 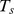 (i.e. the family in our simplified model).
- The neighbors of infected individuals can move without any restriction.

After a time 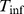 since infection, the infected individual acquires permanent immunity (i.e. develops antibodies), changing the internal degree of freedom 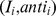 from 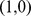 to 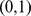, and starting to move again. Each infected individual has her/his own infective period. In particular, we assume the infective period, 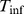, and the stop time, 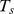, to follow exponential distributions, with the same parameter values adopted in [26] and reported in Table 1. The infectivity is taken to be constant during the disease.

**Table 1:** Parameters of the model fixed a-priori.

** Lattice parameters**	** Value**
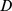	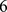
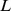	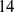
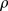	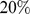
** Epidemiological parameters**	** Value**
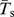	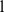 day
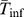	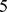 days
** Behavioral change parameters**	** Value**
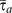	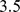 days
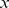	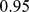
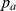	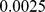

For the typical duration of influenza symptoms and asymptomatic phase see for instance http://www.cdc.gov/h1n1flu/qa.htm.

This simplification corresponds to disregard the effect of the viral load in the infection process, which is indeed entirely ascribed to the immunological status of the susceptible individual. To disregard the viral load in the transmission process is acceptable for highly infective disease, as the influenza pandemic. In any case, the model can be easily upgraded in order to overcome this simplification, introducing a variable infectivity and a susceptible-infector dependent transmission rate.

### Introduction of self-initiated human behavioral changes

In this section, we consider the interplay between human behavioral changes and epidemic spreading. We focus on self-initiated behavioral changes that reduce the susceptibility to the disease without altering the contact patterns among individuals. The only modifications of the structure of the contact network are the adaptive mobility restrictions already included in the baseline model.

Susceptible individuals, which become aware of the risk of infection, adopt a protective behavior in order to reduce the risk of contagion (increased hygienic measures, use of face masks, etc), which may alter the type but not the number of contacts. We introduce a susceptibility reduction factor 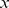 for the aware individuals with respect to the unaware ones. 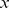 is independent of the age class and equal for each individual. An extra degree of freedom for the awareness status (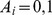) is associated to each individual. After a potentially contagious contact with an infected individual, an aware susceptible individual becomes infected according to the reduced susceptibility 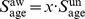, with 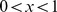. We also introduce a memory effect such that aware individuals lose their awareness after a time 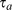, which is exponentially distributed with average awareness time 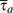 (reported in Table 1), and a truncation of the distribution at 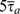.

We consider three different mechanisms of awareness spreading.

- I mechanism (Awareness at local level - Prevalence Based). Individuals that are nearest neighbors of infected symptomatic ones, become aware and acquire state of awareness 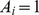. The awareness induces individuals to adopt preventive measures with the effect of reducing their susceptibility to the values 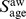. In this case, the information which induces awareness is prevalence based and locally available, according to the classification in [24].
- II mechanism (Awareness at global level - Prevalence Based). The global spread of awareness, due to the news about the progress of the epidemic published by the mass media, or to institutional educational campaigns, induces people to change their behavior. We consider a prevalence based mechanism of awareness spreading, triggered by the globally available information. In particular, at each time 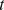, the state of awareness 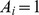 is attributed to a number of randomly chosen individuals, 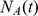, depending on the total number of infected individuals by the following relation: 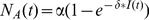
(4)where 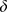 and 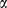 are fitting parameters, and 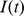 per mil is the fraction of the infected individuals at time 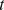. Eq.(4) recalls Eq.(7) of Ref. [17], for 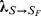, which represents the rate, at which susceptibles acquire awareness in a similar behavior-disease coupled model with a global mechanism of awareness spreading triggered by mass media. Adopting Eq. (4), we obtain that 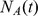 is maximum when 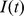 is also maximum. In this way, we implicitly assume that the time evolution of the information spread by the media, is functionally dependent on the time evolution of the number of infective individuals, without any temporary effect of amplification or falsification.

The alert phase lasts until the end of the epidemic.

- III mechanism (Awareness at local level - Belief based). With the “thought contagion”, an aware individual persuades an unaware one to adopt a protective behavior in order to prevent the disease. This contagion mechanism of the state of awareness takes place among nearest neighbors and is, in all respects, similar to the contagion of the infectious disease. This is an example of awareness raising due to a belief based and locally available information. To include this mechanism, we adopt the following procedure: when an unaware individual moves, we check for the status of awareness of the nearest neighbors. If there is one aware nearest neighbor, the individual becomes aware (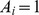) with a certain probability, 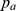. The parameter 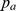 represents the probability of becoming aware after each contact with an aware individual. Thus, in the case of multiple simultaneous contacts with aware individuals, the procedure of “awareness infection” is repeated for each of them. Analogously, when an individual moves, he spreads his state of awareness to the nearest neighbors with the same probability. In any case the update of the awareness state precedes the update of the disease state.

In the following, we consider the case, in which the first mechanism of awareness spreading due to the contact with infected individuals works alone (Scenario A). Then, we discuss the case, in which also the second mechanism of awareness, due to the global spreading of information through the mass media, is added to the first one (Scenario B). Finally, we consider the case in which also the third mechanism of awareness spreading, due to the contagion of fear, is added to the first two mechanisms (Scenario C).

## Results

In Ref. [26], a population with the age class distribution of the Italian one (Table 2) was placed on a lattice, with linear size 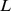 and occupation percentage 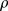. A value of 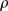 equal to 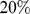 was fixed a-priori in all the studied cases. This value of 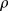 is fixed in order to balance between the average number of nearest neighbors and the mobility of individuals on the lattice. Lattices with different Euclidean dimension 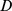 were considered, and 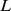 was fixed so that the number of individuals 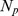 was roughly 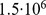 for each 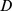 (for 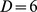, 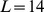). For fixed 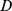 and 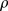, at high enough 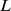, the finite size effects observed by varying 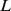 are negligible. For each 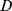, the parameters 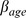 and 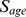 were obtained as the best fit parameters that allow to reproduce the age-specific physical and non-physical daily contact numbers of the Polymod survey, and the peaks of the age specific curves for the H1N1 pandemic in Italy, respectively. The 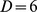 model turned out to be the one that better fit the experimental data (for further details, we refer to Ref. [26]). For this reason, in the present paper, we focus only on the 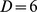 case (which we refer to as reference simulation), and in the following, we fix all the above parameters at the values chosen in Ref. [26] for that case (Tables 2 and 1). As in Ref [26], the simulations are initialized with a density of infected individuals, randomly distributed on the lattice, which is equal to the 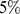 of the density of influenza-like illness (ILI) cases observed at 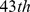 week during the A(H1N1) pandemic in Italy. This is roughly the smallest value of initial infected individuals necessary to activate the spreading of the disease. Below that threshold the infection process immediately fall off. The simulated illness cases, and their errors, are evaluated respectively as mean values, and standard deviations, over 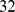 independent processes. Starting with this simulation, we analyze what happens when the three mechanisms of awareness spreading listed in the previous section are included into the model. First, we consider the case, in which the first mechanism of awareness spreading due to the contact with infected individuals works alone (Scenario A). Then, we discuss the case, in which also the second mechanism of awareness, due to the global spreading of information through the mass media, is added to the first one (Scenario B). Finally, we consider the case, in which also the third mechanism of awareness spreading, due to the contagion of fear, is added to the first two mechanisms (Scenario C).

**Table 2:** Age Class Parameters.

Age group	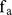	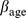	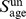	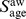
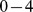	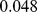	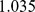	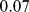	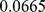
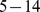	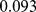	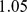	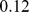	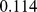
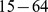	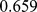	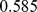	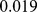	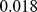
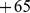	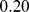	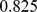	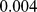	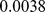

2nd column: distribution of the Italian population in age classes, ISTAT - 2009; 3rd column: age dependent mobility parameters; 4th column: age dependent susceptibilities for unaware individuals; 5th column: age dependent susceptibilities for aware individuals. The susceptibilities of aware individuals 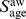 are reduced by a factor of 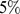 with respect to the unaware 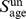.

**Figure 2:**
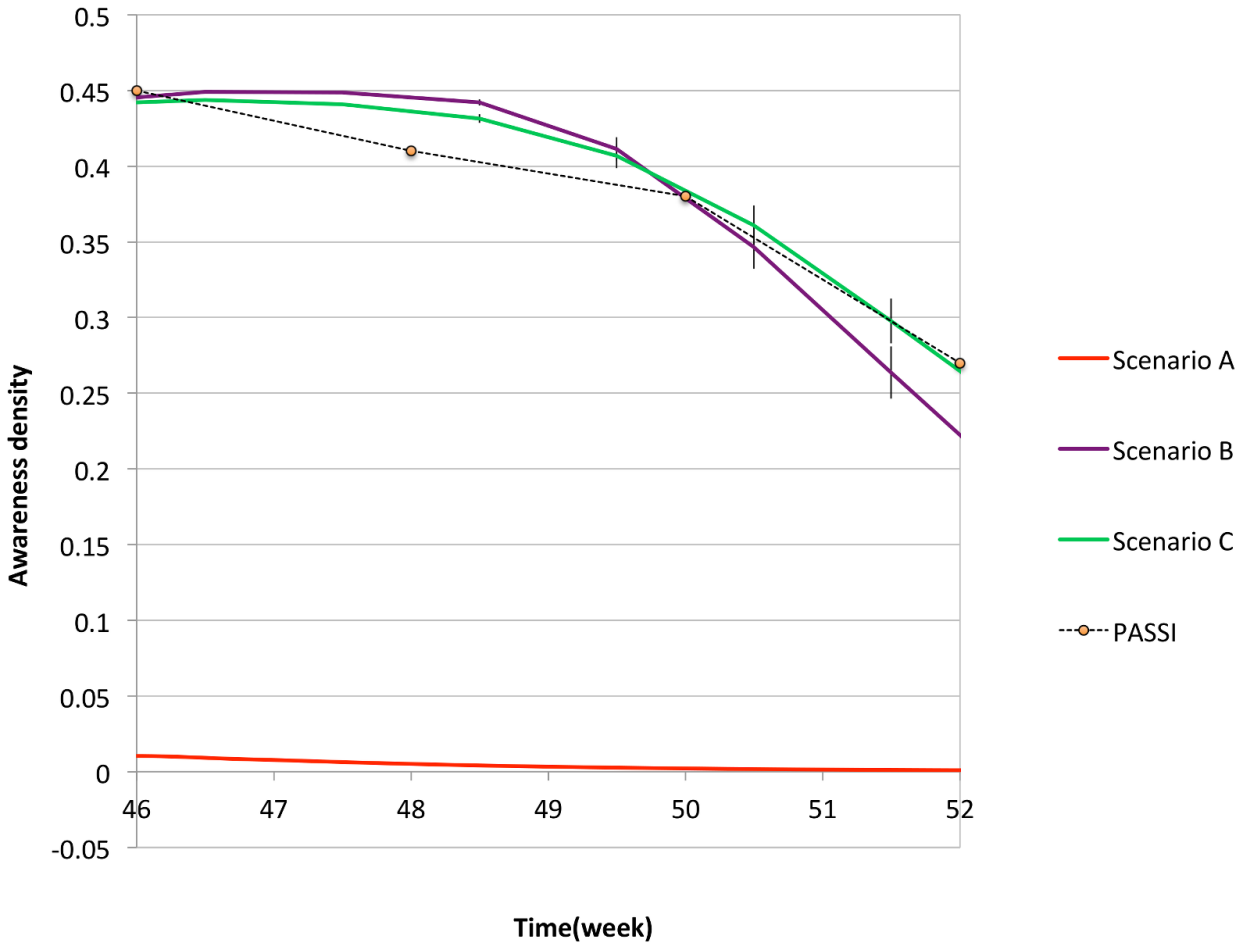
The density of aware individuals in the 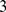 scenarios is compared with the data gathered by the PASSI survey [28], courtesy of the authors.

**Figure 3:**
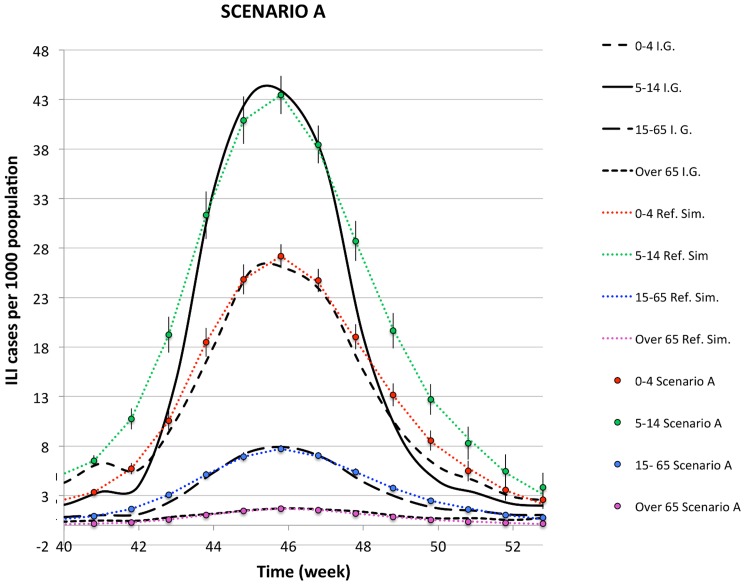
The simulated illness cases by age group in SCENARIO A are compared with the reference simulation and the corresponding epidemiological data for the H1N1 pandemic in Italy during the season 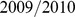, furnished by the sentinel surveillance system Influnet.

**Figure 4:**
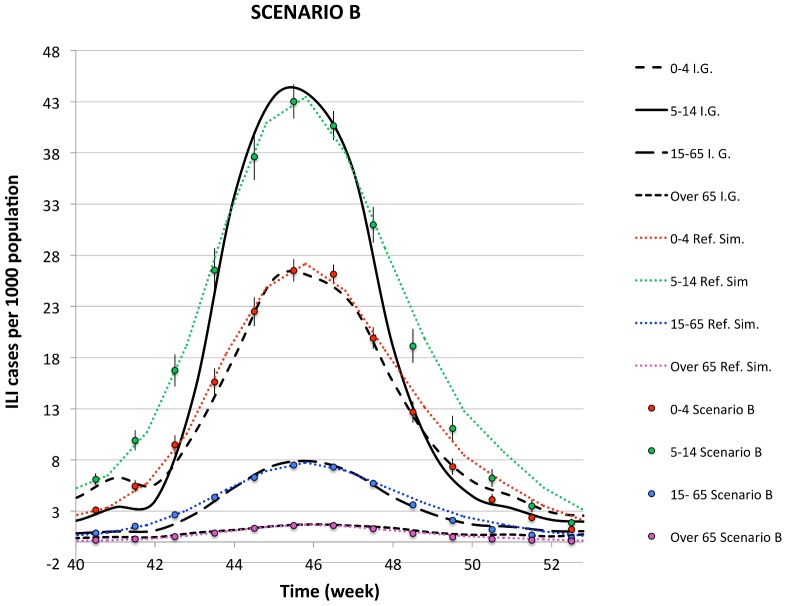
The simulated illness cases by age group in SCENARIO B are compared with the reference simulation and the corresponding epidemiological data for the H1N1 pandemic in Italy during the season 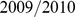, furnished by by the sentinel surveillance system Influnet.

**Figure 5:**
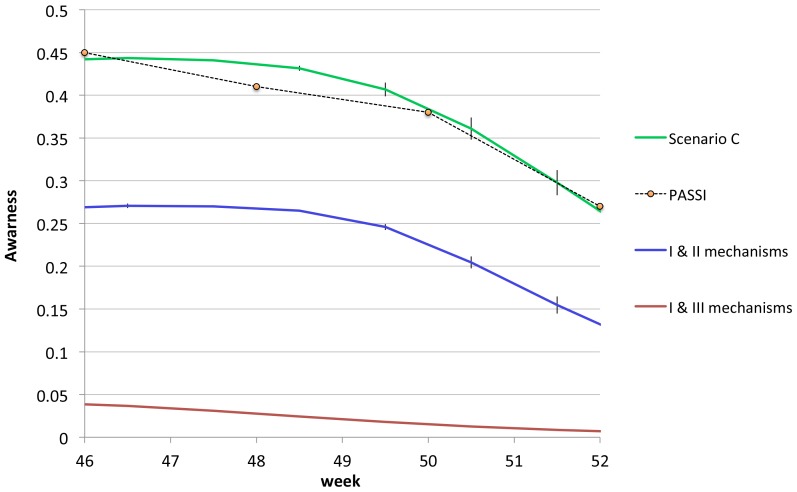
The simulated illness cases by age group in SCENARIO C are compared with the reference simulation and the corresponding epidemiological data for the H1N1 pandemic in Italy during the season 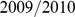, furnished by the sentinel surveillance system Influnet.

In Fig. 2, the density of aware individuals is plotted in the 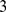 scenarios, starting from the epidemic peak (roughly 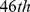 week), while Figs 3, 4, 5 show the comparison of the simulated illness cases by age group 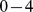, 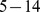, 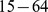, over 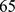 years old, with the corresponding epidemiological data on the ILI cases and the reference simulation, in the 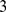 scenarios respectively. In each case, the susceptibilities of aware individuals are reduced by a factor 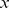, starting from the epidemic peak. We choose a reduction factor 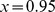, corresponding to a 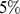 reduction of the susceptibilities (listed in Table 2) of aware individuals with respect to the unaware ones. Such a value is chosen in order to simulate a slight reduction of the susceptibility, as the one expected with the application of basic restrictive hygienic measures. More drastic and efficient measures, as for instance spontaneous vaccination, are not considered.

Let us discuss each scenario separately.

### 

#### Scenario A

In this scenario, awareness is exclusively generated by the contact with symptomatic infected individuals, and the time evolution of the awareness density (red line in Fig. 2) is an outcome of the epidemic spreading. For average awareness time 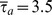 days, as fixed here, the awareness density turns out to be extremely low compared to the PASSI data. Correspondingly, the epidemiological data are indistinguishable from the reference simulation, as shown in Fig. 3. If we increase 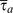 (e.g., from 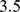 to 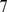 days), only a modest growth in the density of aware individuals is observed, essentially due to the fact that an individual, who becomes aware at a time 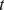, has a higher probability to be still aware after 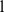 week. However, the overall number of individuals, that acquire awareness during the spreading of the epidemic, does not change, since it depends only on the time evolution of the epidemic. As a consequence, the awareness density obtained with this increased value of 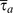 still remains negligible with respect to the one given by the PASSI survey, and the effect on the disease is negligible as well. This is a coherent result within our model, because the contact with infected symptomatic individuals is quite rare, as a consequence of the adaptive mobility rules already present in the baseline model, which on one side prevent symptomatic infected to move and, on the other side, interdict the sites that are nearest neighbors to them. Correspondingly, the effect of the awareness due to this mechanism is unnoticeable, at least for this value of 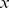. Decreasing the value of the susceptibility reduction factor, 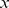, one can see that the corresponding data do not significantly modify, unless one reduces the parameter 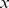 to very small and unrealistic values (at least smaller than 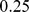).

#### Scenario B

In this scenario the global prevalence-based spreading of awareness, triggered by the mass media and introduced according to Eq. (4), is added to the local mechanism already present in the first scenario. In this case, the parameters 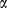 and 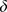 in Eq. (4) can be adjusted in order to fit the PASSI data.

We first perform some simulations by varying 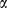 and 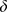 until we locate a small region of the parameter space, in which the number of aware individuals is close enough to the PASSI data. In this region, we construct a fine grid in the 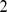 dimensional 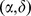 parameter space, run the simulation and evaluate the number of aware individuals in correspondence to each point of the grid and each time 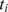 for which PASSI data are available. The final state, adopted to run the epidemic, is chosen as the one that minimizes the 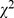: 
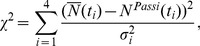
(5)where 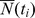 are the simulated number of aware individuals, each obtained as the average value over 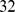 independent processes, 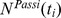 are the corresponding values obtained with the PASSI survey, and 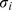 is the standard deviation 
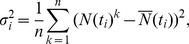
(6)where the sum over 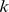 runs over the 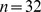 identical and independent processes. In particular, the best fit procedure selects the values of 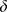 and 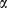 reported in Table 3. The corresponding density of aware individuals is compared to the PASSI data in Fig. 2. The epidemiological curves, plotted in Fig. 4, display a descendant phase, which is quicker than the one in the reference simulation. This is particularly true for the 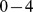 and 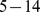 age classes, for which the descendant phase becomes closer to the experimental data.

**Table 3:** Fitting parameters.

Behavioral change parameters	Value
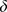 (scenario B)	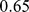
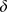 (scenario C)	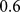
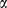 (scenario B)	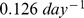
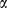 (scenario C)	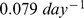

#### Scenario C

This scenario includes all three mechanisms of awareness spreading. Here, the thought contagion has the effect of amplifying the awareness spreading mainly due to the globally available information. In this case, the probability of awareness contagion is a-priori fixed equal to 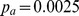. Such a small value is required in order to avoid that the percentage of aware individuals reaches unrealistically high asymptotic values. Again, through a best fit procedure, the values of 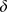 and 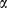 are obtained (see Table 3). In this case we get an even better agreement with PASSI data, as shown in Fig 2, and a corresponding slight improvement in the accordance between epidemiological data and simulated data, as shown in Fig 5.

Comparing Figs 3–5, one can notice that a better reproduction of the awareness data of Ref. [28], is accompanied with a better reproduction of the descendant phase in the epidemiological curves, which become quicker increasing the number of aware individuals. This circumstance confirms the hypothesis formulated in Ref. [26], according to which a possible reason for the disagreement in the descendant phase for the age group 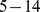 could be the self-initiated health care measures carried out by many Italian families.

**Figure 6:**
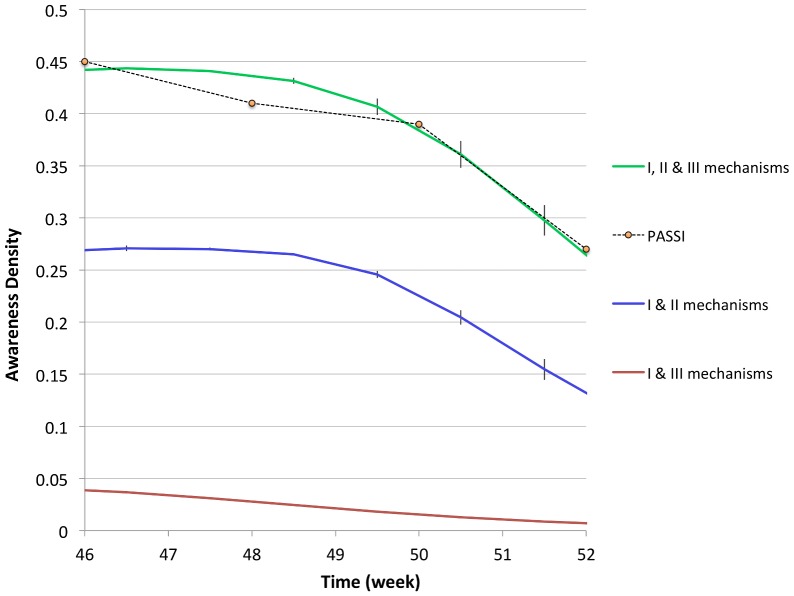
Contributions to the density of aware individuals due to the different mechanisms, compared with the PASSI data for high risk perception [28], courtesy of the authors.

It is also interesting to analyze the role of different mechanisms in reproducing the PASSI awareness plot. As already stated, the first mechanism is completely marginal, thus we concentrate on the comparison between the second and third mechanisms. By keeping fixed the value of 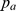 as in scenario C (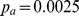), we turn off the second mechanism of awareness spreading, due to the mass media, in order to isolate the contribution of the thought contagion mechanism. In Fig. 6, the density of aware individuals obtained in this case (red line) is compared with that of scenario C, in which all the three mechanisms are active, and PASSI data. We see that, by turning off the mechanism triggered by the mass-media, the density of aware individuals, which is always lower than 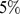, decreases in time and tends to zero. One may wonder if the PASSI data could be reproducible without mass media, by simply increasing the probability of thought contagion 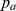. However, it turns out that increasing 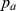 (e.g. 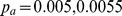, data not shown here) the density of aware individuals still stay lower than the PASSI density plot and, more importantly, it tends to a plateau, whose value is an increasing function of 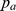. For higher 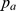 (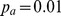), we find that the density of aware individuals reaches an unrealistic plateau of roughly 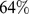 of the population. On the contrary, by turning off the mechanism of thought contagion, and keeping only mechanisms I and II at work, the awareness curve (blue line in Fig. 6) follows from below the same trend of the PASSI one. We conclude that, within our model, the inclusion of the second mechanism of awareness spreading due to the mass media is crucial for obtaining the trend of the PASSI plot.

Summarizing, we find that

- Scenario A does not allow to reproduce a realistic curve for the awareness density, i.e. the PASSI data [28]. This result is consistent with the fact that the contact with a symptomatic infected individual is a rare event in our model, because the adaptive mobility rules prevent symptomatic infected individuals to move, and interdict their nearest neighbor sites. Thus, the spreading of awareness through this mechanism is quite marginal. Consistently, the comparison between the simulated epidemic curves and the reference simulation shows that they are indistinguishable. An appreciable displacement from the reference simulation can only be obtained for unrealistic reductions of the susceptibilities of aware individuals with respect to the unaware ones.
- Both scenario B and C allow to roughly reproduce the PASSI awareness density plot, with the appropriate choices of the fitting parameters 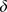 and 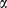 in Eq. (4) (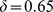 and 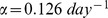 in Scenario B and 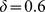 and 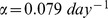 in Scenario C).
- In scenario B and C, the comparison between the simulated epidemic curves and the reference simulation shows that, as expected, increasing the awareness, the descendant phase becomes quicker and quicker and the epidemiological data are better reproduced.
- The comparison between the epidemic curves obtained in scenario B and C (Figs 4 and 5) shows that, in spite of the specific mechanisms, which cooperate to generate the expected awareness, as far as the same density of aware individuals (i.e. the PASSI data) is obtained, the epidemic curves do not significantly differ from each other.
- The simulations realized by turning off the II mechanism of awareness contagion show that the global spreading of awareness triggered by the mass-media is crucial to reproduce the specific trend of the PASSI curve.

## Discussion

In this paper, we propose a very simple model for the epidemic spreading in an age-structured population with dynamic contacts and human behavioral changes. The main purpose is to analyze how the introduction of behavioral responses modifies the spreading of an infectious disease.

In literature there are several papers that treat the interplay between epidemic spreading and self-initiated behavioral changes. Some of them mainly focus on the spreading of awareness through a mechanism of thought contagion (e.g. [16], [31]), others consider the presence of infected individuals in the neighborhood as the main reason for awareness raising (e.g. [14], [32]), others take into account also global information mechanisms (e.g. [17], [33]). The novelty of the present study is that we do not fix a-priori one mechanism of awareness spreading, but consider different scenarios, and compare the simulated data with available experimental data on the risk perception. Thus, the identification of the crucial mechanism comes from the comparison with available data. We choose to reproduce data on awareness of the Italian population about the risk of contagion during the 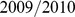 A(H1N1) epidemic outbreak, gathered by the behavioral risk factor surveillance system in Italy (PASSI). Then, we analyze how the presence of awareness in the population changes the progression of the epidemic. We find that, improving the accordance between the simulated and the experimental data on awareness, a better agreement between simulated and experimental epidemiological data is also achieved. In particular, we show that the local and prevalence based mechanism of awareness, due to the contact with a symptomatic infected individual, is a marginal mechanism, in our model, whereas the two important ones are the prevalence based global mechanism due to the information spread by the mass-media/educational campaigns, and the one due to the thought contagion among individuals. We have also shown that, excluding the global information spreading mechanism, a realistic trend for the awareness density cannot be reproduced.

There are some limitations which affect this study, besides those concerning the dynamic and epidemic model, already discussed in Ref. [26]. In particular, the functional form assumed for the global spreading mechanism, given in Eq. (4), establishes a functional relation between the awareness spread by global information and the disease prevalence. With this choice, we disregard the effect of possible media misrepresentation or temporary amplification of the perceived risk of contagion [34], not correlated to the disease prevalence. In a future work, we propose to further analyze the data on the media coverage (frequency, duration and space on the Italian television and newspapers, dedicated to the H1N1), and make a correlation analysis among these data, the perceived risk of contagion [35] and the disease prevalence data [36], in order to check the functional form of Eq. (4). This analysis could help in identifying further global mechanisms of information spreading, independent on the disease prevalence, that could have had a role in raising the awareness of the risk of contagion.

Another limit of our analysis is that the data furnished by the PASSI survey start from the epidemic peak. This is the reason why we choose to study the effects of awareness starting from the epidemic peak too. Indeed, an extrapolation of data at time before the peak cannot be trusted to describe how the risk was perceived from the beginning of the epidemic in Italy. In fact, as discussed in Sect. 0, the alarms launched by the Italian mass media was an almost discontinuous process, with the alternation of periods of scaremongering and skepticism. The content of the news also changed in time, moving from international aspects, during spring and summer, to the local context, during the epidemic peak. In this scenario, a declining trend of the risk perception, starting from the initial phase of the epidemic, seems to be quite unrealistic, if compared with the newspaper coverage and content. It is instead reasonable to expect that, even if the knowledge about the pandemic and the awareness of the main hygienic measures to control the epidemic spreading were widespread among the population before the peak, the risk perception increased when people felt the infection “close to home”, i.e. when they perceived the concrete risk of being infected. Similar analysis on the correlation between media coverage and risk perception in other European countries showed that the initial peak of information, during the earliest stage of the outbreak, was typically not accompanied by a correspondingly high level of worry (see for instance [37]). Furthermore it has been shown that the correlation between media coverage and risk perception increases with the geographical proximity [38] and when the media report local, rather than international, news [35].

A further limit comes from the fact that, as previously emphasized, we only concentrate on behavioral changes that alter the parameters of the epidemic (i.e. the susceptibility to the disease) and do not consider the case, in which the effect of the awareness is a reduction in the number of contacts. Even if in principle it is hard to say which kind of behavioral response is most likely to be assumed by aware individuals, in [37] it has been shown that the exposure to the media campaign on the risk of contagion in UK increased the perceived efficacy of hygiene behaviors and decreased the perceived efficacy of social distancing and avoidance behavior. We believe that the media campaign in Italy had a similar outcome, as confirmed also by the PASSI survey. Furthermore, introducing a social distancing mechanism would lead to a structural modification of the network of contacts. However the contact patterns in the present model are fixed in order to reproduce the average daily contact numbers per age-class furnished by the Polymod large-scale survey. Polymod data were collected in absence of an epidemic outbreak, so these data do not allow to reconstruct the modifications that occur during an epidemic. If data on contact patterns among individuals during an outbreak were also available, this could allow to include social distancing effects in our model.

Given the low coverage of pandemic vaccine that has poor protective effects against the epidemic (e.g. [39]), we decided not to include the vaccination measure in the present model. However, this process was already discussed in [26] and could be straightforwardly included also in this behavioral - disease model.

Finally, it would be very interesting to study the behavior of the system by varying the parameters, in order to analyze how different parameters and mechanisms influence the epidemic size, the timing of the epidemic, and the appearance of multiple waves. We leave such analysis to future work.
